# How are patients managing with the costs of care for chronic kidney disease in Australia? A cross-sectional study

**DOI:** 10.1186/1471-2369-14-5

**Published:** 2013-01-10

**Authors:** Beverley M Essue, Germaine Wong, Jeremy Chapman, Qiang Li, Stephen Jan

**Affiliations:** 1 The George Institute for Global Health, University of Sydney, Missenden Road, PO Box M201, Sydney, NSW, 2050, Australia; 2 Children’s Hospital at Westmead, Centre for Kidney Research, Westmead Hospital, The Research Building, Level 2, Sydney, NSW, 2145, Australia; 3 Westmead Hospital, Acute Interventional Medicine and Renal Services, Darcy Road, Westmead, Sydney, NSW, 2145, Australia

**Keywords:** Chronic kidney disease, Economic hardship, Out-of-pocket costs, Australia

## Abstract

**Background:**

Chronic kidney disease (CKD) poses a financial burden on patients and their households. This descriptive study measures the prevalence of economic hardship and out-of-pocket costs in an Australian CKD population.

**Methods:**

A cross-sectional study of patients receiving care for CKD (stage III-V) in Western Sydney, Australia using a structured questionnaire. Data collection occurred between November 2010 and April 2011. Multivariate analyses assessed the relationships between economic hardship and individual, household and health system characteristics.

**Results:**

The study included 247 prevalent CKD patients. A mean of AUD$907 per three months was paid out-of-pocket resulting in 71% (n=153) of participants experiencing financial catastrophe (out-of-pocket costs exceeding 10% of household income). Fifty-seven percent (n=140) of households reported economic hardship. The adjusted risk factors that decreased the likelihood of hardship included: home ownership (OR: 0.32, 95% CI: 0.14-0.71), access to financial resources (OR: 0.24, 95% CI: 0.11-0.50) and quality of life (OR: 0.12, 95% CI: 0.02-0.56). The factors that increased the likelihood of hardship included if income was negatively impacted by CKD (OR: 4.80, 95% CI: 2.17-10.62) and concessional status (i.e. receiving government support) (OR: 3.09, 95% CI: 1.38-6.91). Out-of-pocket costs and financial catastrophe were not found to be significantly associated with hardship in this analysis.

**Conclusions:**

This study describes the poorer economic circumstances of households affected by CKD and reinforces the inter-relationships between chronic illness, economic well-being and quality of life for this patient population.

## Background

Chronic kidney disease (CKD) management imposes a substantial financial burden on both health systems and on patients and their households. In 2004-05 the total health expenditure for CKD exceeded AUD$898.7 million in Australia - 1.7% of the health care budget [1] and similar levels of expenditure are reported for other OECD countries [2,3]. Against this is a background of increasing health expenditure in the form of out-of-pocket payments [4]. Supportive care provided to Australian patients starts at around AUD$50,000 per annum for dialysis care [5] and while out-of-pocket expenses are only a small proportion of the total cost of care, their impact can be severe. For example, in a 2006 study of Australian patients receiving dialysis care in Victoria either in a community location or at home, patients paid an average of AUD$1,237 and AUD$480 out-of pocket annually respectively [6]. These expenses occur at a time when there are interruptions to paid employment and earlier than expected departures from the workforce for the patient and often a family carer [7].

Most studies that estimate out-of-pocket costs only quantify direct costs for treatment and medications [6], overlooking the often substantial costs that are associated with self-management, including: medically related transport, home-care assistance, illness-related home modifications (e.g. for home dialysis set-up) and assistive devices. In Australia, the direct costs of medical care are at least partly covered by health insurance or the state (See overview of the Australian health system below). However, self-management expenses are usually paid by patients so previous studies likely under-estimate the true out-of-pocket costs associated with CKD. Furthermore, few studies have quantified the burden more broadly to patients and households in terms of economic hardship and financial distress. In other chronic disease patient populations high rates of economic hardship have been reported based on a reported inability to maintain important living expenses [8-10], medical care costs [11] and with this, compromised adherence to medical care and quality of life [1,12]. As CKD disproportionately affects lower socioeconomically disadvantaged individuals it can reasonably be expected that this patient population is also at risk of economic hardship.

**An overview of the Australian health and social welfare systems**[4]

The Australian government operates a national health insurance scheme known as Medicare, which subsidises non-public outpatient medical treatment and medications. Medicare rebates a portion of the costs of medical, nursing and allied health services on a fee-for-service basis. Some medical services can be bulk-billed, meaning that the provider charges the same amount as the Medicare rebate, resulting in no out-of-pocket expense. Prescription pharmaceuticals that are approved and listed on the Pharmaceutical Benefit Scheme are also subsidised and individuals are responsible for contributing a co-payment. The Australian government administers safety net programs to cap individual out-of-pocket costs for medical services and prescription pharmaceuticals. In addition, the State and Territory governments operate a public hospital system, which provides inpatient services free of charge. They also provide some support for treatment and management aids and services (e.g. transport and appliances) and this is usually means tested. Private health insurance is available for supplementary services such as private hospital treatment, dental services and allied health services and is heavily supported by government subsidies and tax rebates.

The Australian government also operates a separate national welfare system known as Centrelink. Income support through Centrelink is available for age pensions based on means and asset tests and disability pensions based on functional capacity. Pensioners, also referred to as concessional patients, are entitled to receive a number of concessions and subsidies for living, medical and pharmaceutical expenses and are more likely to receive bulk-billed services.

This descriptive study had the following two aims: 1) to quantify the economic burden among patients with CKD and identify factors that are associated with economic hardship; and 2) to quantify out-of-pocket spending associated with CKD care.

## Methods

### Setting and participants

We recruited English-speaking individuals currently receiving care for CKD III-V in Western Sydney, Australia. An opt-in invitation and a study questionnaire were mailed to a cross-section of individuals, identified by convenience sampling by clinic staff, across the spectrum of illness, including those receiving renal replacement therapy. Individuals either self-administered the questionnaire or completed it with the assistance of a researcher over the phone. The questionnaire was re-sent to all non-respondents six weeks after the initial mailing and the remaining non-respondents at 10 weeks were re-contacted by telephone with a further reminder.

In addition, all English-speaking individuals receiving dialysis therapy in the three community-based dialysis facilities that served the Western Sydney area were also invited to participate. Individuals who had already completed the questionnaire by post were not invited to participate again. Clinical staff at each centre provided information about the study to those who were interested and individuals either self-completed the questionnaire or completed it with the assistance of a researcher.

The return of a completed questionnaire by post was understood to imply consent to participate in the study. Participants who completed the questionnaire in person with the assistance of a researcher provided written informed consent. This study was approved by the Human Research Ethics Committees of the Sydney West Area Health Service (HREC 2008/5/4.14 (2794)) and the University of the Sydney (12623).

### Study questionnaire

The questionnaire was developed based on the authors’ previous work [9,13,14] and included questions that were drawn from existing validated tools [15-18] and covered the following domains: demographics; medical history; quality of life; self-reported chronic illness and disability, household economic circumstances and social connection (i.e. the number of social contacts). The questionnaire domains and variables are described in Additional file 1: Table S1.

### The economic burden of CKD

The economic burden of CKD was measured using the following outcomes: 1) household economic hardship (hardship hereafter) and 2) out-of-pocket costs on medical and health-related expenses.

Hardship was measured using a series of questions about financial stress (e.g. failure to pay basic living and medical expenses) and the use of dissaving actions in the previous 12 months. Dissaving behaviour is any action where spending is greater than income thereby reducing already accumulated savings or leading to borrowing to finance the expenditure [17]. Hardship was constructed as a dichotomous variable where a reported inability to make any of the payments posed or the use of a dissaving action was classed as a case of hardship.

Participants were asked about their out-of-pocket costs in the past three months for the following expenses: prescription and non-prescription medications; medical appointments; hospitalisations; medical tests; medically-related transportation (including: ambulance; public, private and subsidised transport); home and self-care assistance; medical equipment and supplies; illness-related home modifications and special food required for an illness-related diet. The burden of out-of-pocket costs was calculated as total out-of-pocket expenditure in the past three months as a proportion of the household’s equivalised income for the same quarter. Equivalised household income (income hereafter), was calculated using the Organisation for Economic Cooperation and Development’s equivalence scales [19], which make adjustments to actual income to account for households of different size and composition. An out-of-pocket burden greater than 10 percent was defined as financial catastrophe [20].

### Statistical analysis

We conducted descriptive analyses of frequencies for each component of hardship and means, medians and distributions for out-of-pocket spending. Bivariate analyses, using the chi-square test and the independent *t*-test, were used to compare participants with and without hardship for categorical and continuous variables respectively. Given the positive skew of the cost data, the Mann-Whitney U and Kruskal-Wallis one-way ANOVA were used to evaluate differences in median spending in the sample, by stage of illness and by hardship status. Logistic regression was used to identify the factors that were associated with hardship, beginning with a saturated model that included all potential explanatory variables that were associated with hardship at the level of *P* <0.25 in the univariate analysis as well as the variable for age. Variables were assessed individually for significant contribution to the overall model (*P*< 0.05) manually. Effect modification was checked between variables in the model to identify interactions that were significant at the level of *P*<0.01. The Hosmer and Lemeshow goodness-of-fit test was used to check the fit of the final model. Data analysis was conducted using SAS version 9.2.

## Results

### Recruitment and participant characteristics

**Figure 1:**
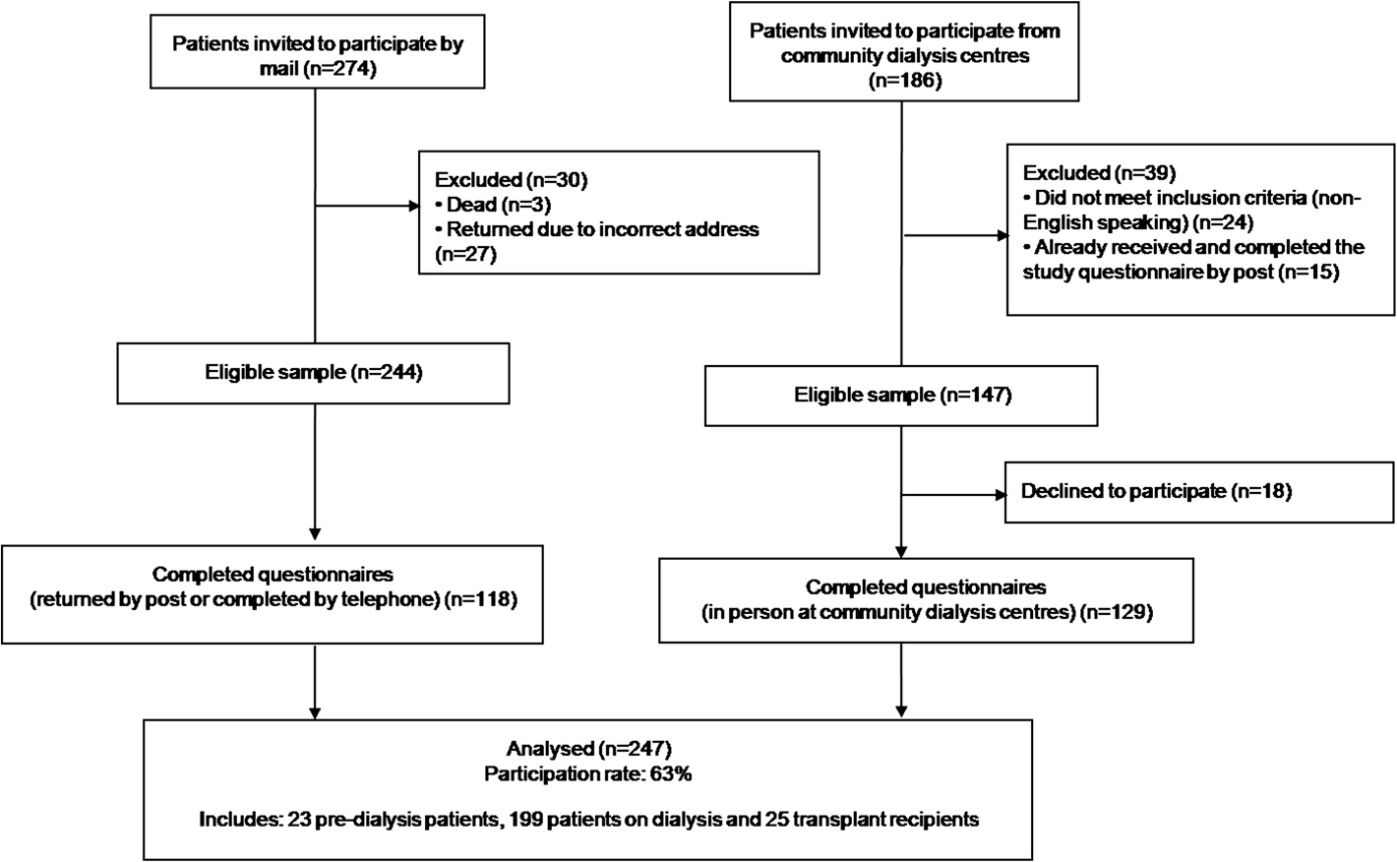
Flow of participants into the study.

This study achieved a participation rate of 63%, n=247 (Figure 1). The sample included 23 patients with CKD who were not yet receiving dialysis (pre-dialysis participants hereafter), 199 participants receiving dialysis therapy, dialysing either at home (n=70) or in a community centre (n=129) and 25 transplant recipients. The mean age of participants was 59 (SD:15) years, 43% were female (n=107) and 65% (n=161) were married or living with a partner (Table 1). Participants reported an average of three comorbid conditions and hypertension (69%, n=170) and diabetes (40%, n=99) were most commonly reported. The average quality of life score (score between zero and one – a higher score signifies better quality of life) was 0.64 (SD: 0.25) and this did not differ significantly by the stage of CKD. Most participants were out of the workforce, either retired (n=121) or unemployed (n=76) and 74% (n=183) reported receiving some form of government support (e.g. income assistance, concessions or subsidies for living expenses) in the past year. In addition, 37% (n=91) of participants had private health insurance.

**Table 1:** Participant characteristics overall and by hardship status

	**All participants (n=247)**	**Hardship (n=140)**	**No hardship (n=107)**	***P-value***
Mean age (years)	59 (SD:15)	56 (SD:14)	62 (SD:15)	0.001
Gender (Females)	107/246 (43%)	59 (42%)	48 (45%)	0.71
Culturally and linguistically diverse	85/246 (35%)	54 (39%)	31 (29%)	0.13
Married / defacto	161/247 (65%)	92 (66%)	69 (64%)	0.84
Home ownership (yes)	101/246 (41%)	34 (24%)	67 (63%)	<0.0001
Employment status				<0.0001
Employed, full-time	31/245 (13%)	12 (9%)	19 (18%)	
Employed, part-time (<20hrs/week)	17/245 (7%)	11 (8%)	6 (6%)	
Unemployed	76/245 (31%)	60 (43%)	16 (15%)	
Retired	121/245 (49%)	56 (40%)	65 (61%)	
Education				0.95
None	7/244 (3%)	4 (3%)	3 (3%)	
Primary and /or secondary school	132/244 (54%)	74 (53%)	58 (55%)	
Tertiary	105/244 (43%)	61 (44%)	44 (42%)	
Income (AUD$)				0.39
Under $379 per week	155/247 (63%)	88 (63%)	67 (63%)	
$380–$579 per week	24/247 (10%)	14 (10%)	10 (9%)	
$580–$769 per week	18/247 (7%)	9 (6%)	9 (8%)	
$770–$959 per week	12/247 (5%)	9 (6%)	3 (3%)	
>$960 per week	14/247 (6%)	10 (7%)	4 (4%)	
Don’t know/rather not answer	24/247 (10%)	10 (7%)	14 (13%)	
Co-morbidity (number)	3/247 (SD:2)	3 (SD:2)	2 (SD:2)	0.10
Cancer	33/247 (13%)	22 (16%)	11 (10%)	0.21
Diabetes	99/247 (40%)	53 (38%)	46 (43%)	0.41
Hypertension	170/247 (69%)	105 (75%)	65 (61%)	0.02
Depression	65/247 (26%)	41 (29%)	24 (22%)	0.23
Cardiovascular disease	84/247 (34%)	45 (32%)	39 (36%)	0.48
Current treatment type				0.53
Pre-dialysis	23/247 (9%)	11 (8%)	12 (11%)	
Dialysis	199/247 (81%)	113 (81%)	86 (80%)	
Home	71/199 (36%)	43 (38%)	28 (33%)	0.42
Community location	128/199 (64%)	70 (62%)	58 (67%)	
Transplant recipient	25% (10%)	16 (11%)	9 (8%)	
Mean time on dialysis	5.7 (SD:5.8)	5.8 (SD:5.4)	5.6 (SD:6.3)	0.83
Mean time since diagnosis, years	12.7 (SD:11.8)	12.0 (SD:11.6)	13.7 (SD:12.2)	0.30
Quality of life^♦^, EQ5D[15]	0.64 (SD:0.25)	0.59 (SD:0.23)	0.70 (SD:0.25)	<0.001
Pre-dialysis	0.62 (SD:0.26)	0.57 (SD:0.23)	0.66 (SD:0.29)	0.42
Dialysis	0.64 (SD:0.24)	0.59 (SD:0.23)	0.70 (SD:0.25)	0.002
Transplant	0.66 (SD:0.26)	0.59 (SD:0.26)	0.77 (SD:0.23)	0.12
Need assistance with ADLs	150/247 (61%)	98 (70%)	52 (49%)	<0.001
Family carer	102/243 (42%)	70 (50%)	32 (30%)	0.003
Number of social encounters (per week)				0.01
0	72/247 (29%)	42 (30%)	30 (28%)	
1-3	127/247 (51%)	80 (57%)	47 (44%)	
4-6	23/247 (9%)	11 (8%)	12 (11%)	
>6	25/247 (10%)	7 (5%)	18 (17%)	
Catastrophic spending (out-of-pocket spending >10% of income)	153/216 (71%)	85 (67%)	68 (76%)	0.13
Mean out-of-pocket spending (AUD$/quarter)*	907 (SD:1070)	930 (SD:1123)	876 (SD:997)	0.18
Receiving any concessions and /or subsidies	183/247 (74%)	115 (82%)	68 (64%)	0.001
Income negatively impacted by illness	142/247 (58%)	105 (75%)	37 (35%)	<0.0001
Private health insurance	91/247 (37%)	40 (29%)	51 (48%)	0.002
Able to pay AUD$2000 for something important in a week (access to financial resources)	125/247 (51%)	42 (30%)	83 (78%)	<0.0001

Data are presented as either mean (SD=standard deviation) or count (%=proportion).

^♦^n=229, missing quality of life data for n=18.

*Difference in median spending assessed using non-parametric statistical tests.

AU$1=US$1=EU€0.73=BGP£0.62; January 2011.

Compared to the treated incident renal population in Australia, our sample had a significantly higher proportion of working-aged adults (45-64 years) (Additional file 1: Table S2). The study population may also have been less sick, given the significantly lower prevalence of co-morbid lung disease and cardiovascular disease, and the significantly higher quality of life among dialysis patients. Participants also reported a higher socioeconomic status, signified by higher proportions of home ownership, health insurance coverage and individuals in paid work. However, when compared with the Australian population as a whole, our population of prevalent CKD patients was generally less well off, with more people out of the workforce and lower health insurance coverage, which is consistent with the characteristics of chronically ill patients in Australia [20].

### Hardship associated with CKD

**Figure 2:**
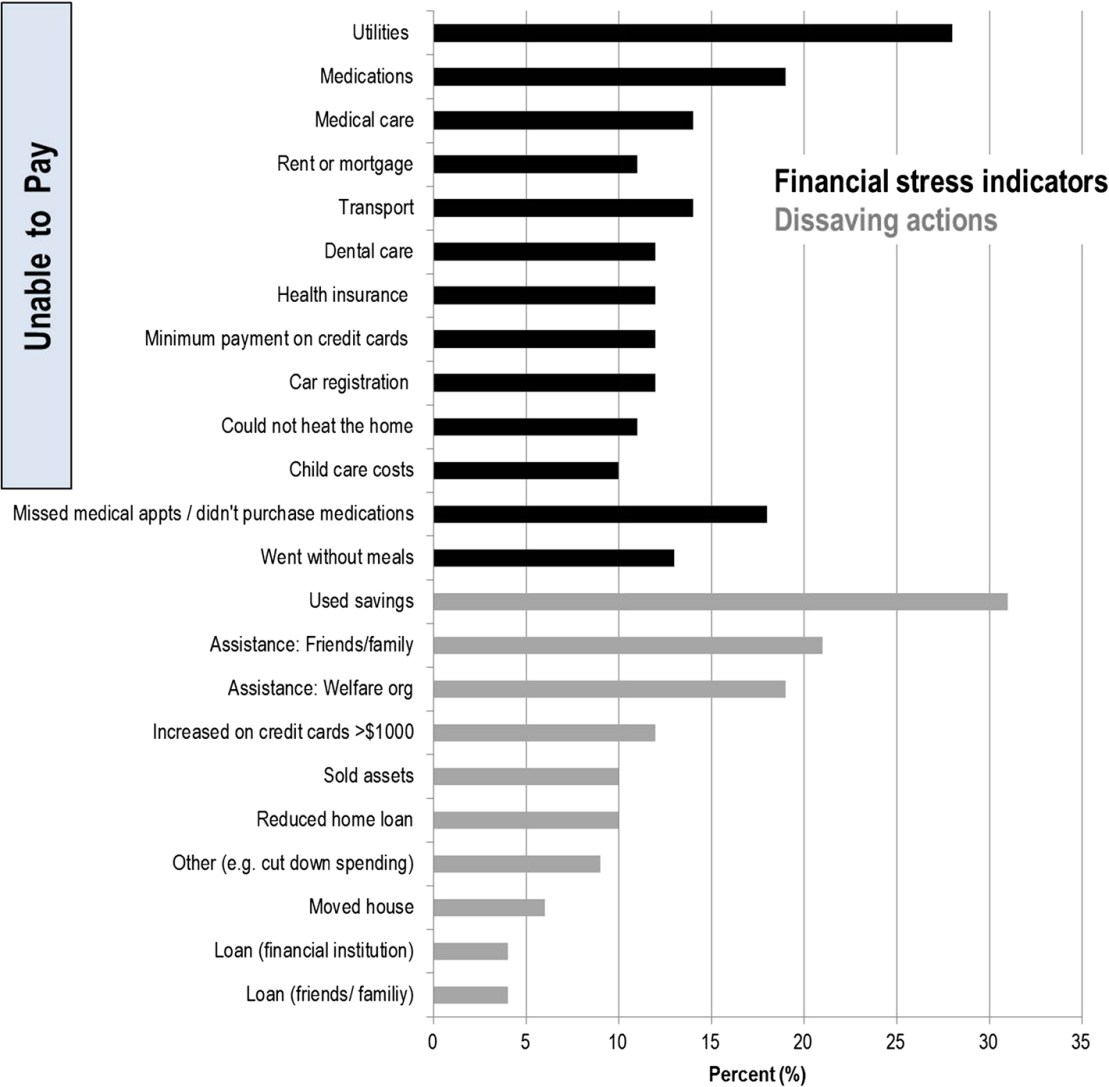
Proportion of participants reporting each indicator of hardship.

Fifty-seven percent of participants (n=140) had hardship (Figure 2). Paying for utility bills posed the greatest burden with 28% (n=69) of participants reporting an inability to pay a gas, electricity or telephone bill in the previous 12 months. Nineteen percent (n=46) were unable to pay for medications, 14% (n=35) were unable to pay co-payments to attend medical appointments and 18% (n=44) reported missing medical appointments or failed to fill prescriptions in the previous year because they were short of money. Drawing on long-term savings was the main dissaving action reported (31%, n=76).

From the multivariable model, after adjusting for age, participants who owned their home (OR: 0.32, 95% CI: 0.14-0.71), had access to financial resources (OR: 0.24, CI: 0.11-0.50) and had higher quality of life (OR: 0.12, CI: 0.02-0.56) were less likely to have hardship (Table 2). The number of social encounters per week also had a non-significant protective effect. The factors that increased the odds of experiencing hardship included: if household income was negatively impacted by CKD (e.g. due to early departure from the workforce or change in employment circumstances) (OR: 4.80, CI: 2.17-10.62) and concession status (OR: 3.09, CI: 1.38-6.91). There was no evidence of effect modification among the variables and the final model fit the data well (χ_HM_^2^=4.48, p=0.81).

**Table 2:** Unadjusted and adjusted logistic regression models of correlates of economic hardship

**Covariates**	**Unadjusted OR (95% CI)**	***P*-value**	**Adjusted**^**a **^**OR (95% CI)**	***P*-value**
Age	0.97 (0.95–0.99)	<0.001	0.99 (0.96–1.02)	0.32
Home ownership (yes)	0.19 (0.11–0.32)	<0.0001	0.32 (0.14–0.71)	0.005
Quality of life	0.14 (0.04–0.43)	<0.001	0.12 (0.02–0.56)	0.007
Number of social encounters (per week)		0.02		0.01
0	1.00		1.0	
1-3	3.60 (1.34–9.69)		1.90 (0.79–4.57)	
4-6	4.38 (1.70–11.25)		0.58 (0.16–2.11)	
>6	2.36 (0.71–7.80)		0.31 (0.09–1.09)	
Receiving concessions and subsidies for living and medical expenses	10.57 (1.47–14.74)	0.001	3.09 (1.38–6.91)	0.006
Income impacted by illness (due to early departure from work or change in employment)	5.68 (3.27– 9.86)	<.0001	4.80 (2.17–10.62)	0.0001
Access to financial resources (could pay AUD$2000 in a week for something important)	0.12 (0.07–0.22)	<.0001	0.24 (0.11–0.50)	0.0001

^a^ Hosmer and Lemeshow goodness-of-fit test; : *χ*^2^: 4.48, p=0.81.

The adjusted model was built manually and included all variables associated with hardship at the level of *P* <0.25 in univariate analysis (shown in Table 1).

### Relationship between out-of-pocket costs and hardship

**Figure 3:**
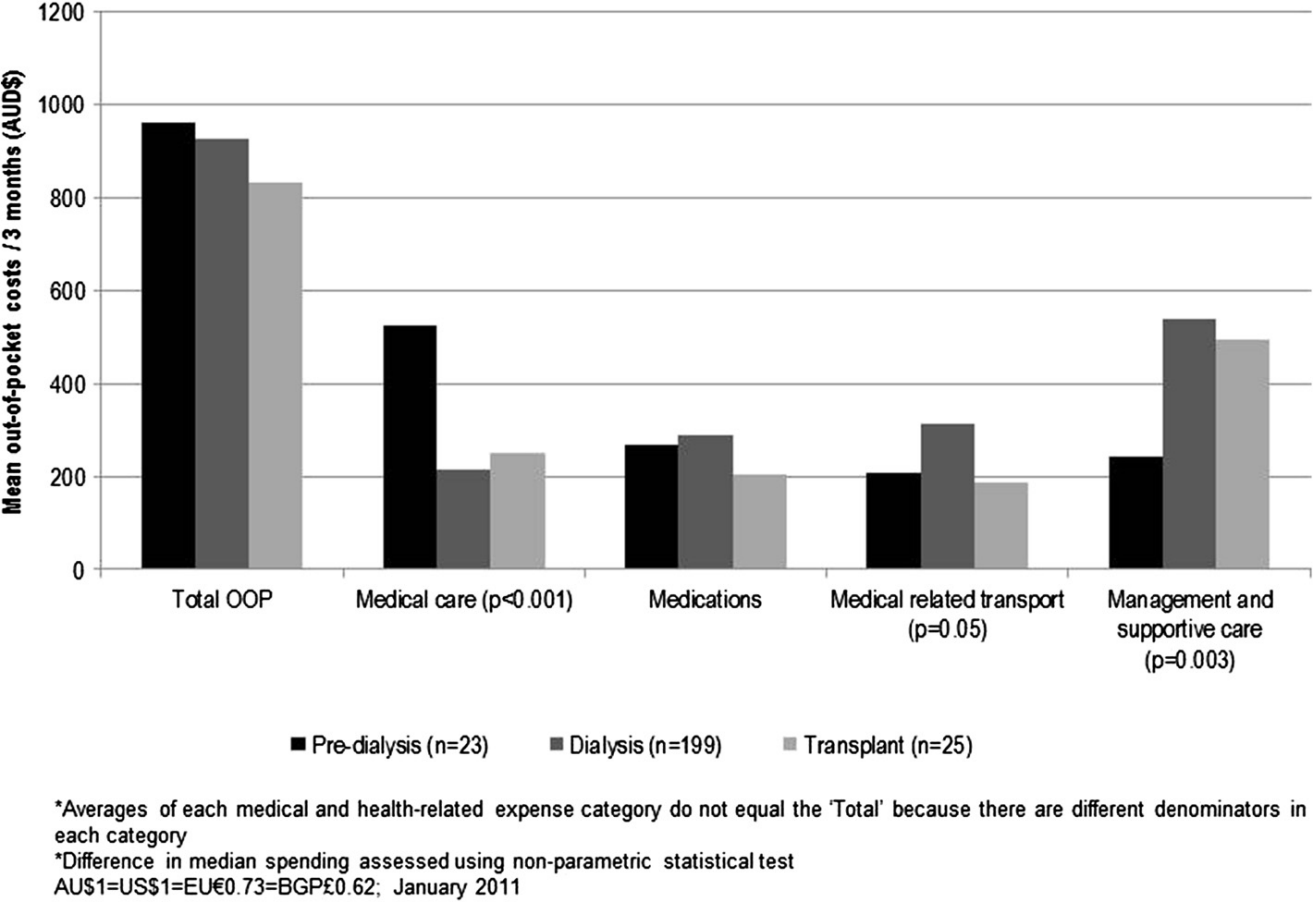
Mean out-of-pocket costs in the previous three months for each medical and health-related expense category by stage of illness.

Similar mean out-of-pocket costs were reported for pre-dialysis, dialysis and transplant care – AU$961 (SD:AU$1211), AU$927 (SD:AU$1089) and AU$831 (SD:AU$803) respectively per quarter (AU$1=US$1=EU€0.73=BG£0.62; January 2011). At this level of spending, 153 (71%) participants experienced financial catastrophe. In terms of the sources of out-of-pocket costs, pre-dialysis participants spent the most out-of-pocket in the previous three months on medical care ($\overset{―}{\text{x}}$=AU$527, SD:AU$1228) (Figure 3). Median spending in this category was significantly higher than that of dialysis and transplant participants (*χ*^2^= 22.33, p<0.0001). Participants receiving dialysis care and those with a transplant both spent the most on management and supportive care in their home, AU$538 (SD:AU$900) and AU$495 (SD:AU$663) respectively, which included: home and self-care assistance; medical equipment; illness-related home modifications and illness-specific diets. Large out-of-pocket costs on illness-related home modifications (>AU$1000) were reported by a small number of transplant (n=2) and home dialysis (n=5) participants. When these cases were removed from the analysis, mean spending in this category was reduced to AU$374 (SD:AU$449) for those on dialysis and AU$250 (SD:AUD$156) for transplant recipients. Finally, participants receiving dialysis also reported significantly higher out-of-pocket spending on medically related transport $\overset{―}{\text{x}}$=AU$312, SD:AU$298; *χ*^2^=6.03, p=0.05).

The multivariable logistic regression model described above did not identify out-of-pocket costs as a significant determinant of hardship. Table 3 summarises the out-of-pocket costs for each medical and health-related expense category. We found similar levels of spending across all cost categories between those with and without hardship, indicating that those with hardship, despite having less financial resources available to them, maintained levels of expenditure comparable to those not experiencing hardship.

**Table 3:** Summary of out-of-pocket costs by hardship status (out-of-pocket costs in the previous three months)

**Out-of-pocket cost category**	**n**	**Minimum**	**Maximum**	**Mean (SE)**	**Mean difference**^**a**^	***P***^**b**^	**Median (IQR)**	***P***^**c**^
All								
Hardship	99	15	5250	876 (100)	54	0.7	655 (800)	0.2
No hardship	136	32	9460	930 (96)			540 (720)	
Medical care								
Hardship	100	0	6150	280 (84)	76	0.5	40 (200)	0.7
No hardship	82	0	5250	204 (74)			53 (150)	
Medications								
Hardship	128	20	2000	304 (27)	57	0.08	200 (280)	0.6
No hardship	100	0	1200	247 (18)			200 (195)	
Transport*								
Hardship	112	0	1800	268 (26)	-60	0.1	200 (304)	0.2
No hardship	87	0	1200	328 (33)			240 (380)	
Management and supportive care								
Hardship	55	15	6430	552 (129)	94	0.6	265 (500)	0.8
No hardship	33	6	2710	458 (113)			230 (350)	

AU$1=US$1=EU€0.73=BGP£0.62; January 2011.

SE: Standard error; IQR: Interquartile range.

* Medically related transport.

^a^ Mean difference: $\overset{―}{\text{x}}$ (hardship) - $\overset{―}{\text{x}}$ (no hardship).

^b^ Difference in means tested using the independent *t*-test.

^c^ Difference in distribution of medians tested using the Mann Whitney *U* test.

## Discussion

This is the first study to comprehensively measure in patients with CKD in Australia the total out-of-pocket costs and the extent of hardship that are associated with the care of CKD. The prevalence of hardship reported in this study is lower than that found for other chronically ill patient populations in the same region [9]. However, it lies well above the levels that tend to be seen in the Australian population [17] and is consistent with rates of hardship in other chronically ill populations internationally [10,21]. In addition, the burden of out-of-pocket costs found in this study, a mean of AUD$907 in the previous three months is far greater than estimates found in other studies [6], reinforcing wider concerns about the extent to which the Australian health care system relies on individual contributions to fund health care. Individual spending on health care in Australia is high by international, high-income country standards. In a recent Commonwealth Fund survey of 11 high-income countries, the incidence of out-of-pocket costs exceeding US$1000 in the previous year among individual participants was 21% in Australia – behind only the United States (35%) and Switzerland (25%) [22]. The current study makes an important contribution to the literature in this area by improving our understanding of the poorer economic circumstances of households affected by chronic illness and identifying the financial stressors that increase the likelihood of hardship in a CKD population.

We found high levels of out-of-pocket costs despite Australia having a comprehensive social health insurance system that is seen to be universal. Previous studies show out-of-pocket costs for treatment can pose serious barriers to adherence to recommended medical care [4,10,23,24], especially for those who are socioeconomically disadvantaged. Patients with hardship were spending similar amounts out-of-pocket overall and in each medical and health related expense category across the stages of CKD. Patients with low means often prioritise paying for medical care over other important expenses [11]. The impact of large out-of-pocket costs for patients with low financial resources can be severe. We found 13% of participants reported going without meals, 11% were unable to heat their homes, 12% increased the amount owing on their credit cards by greater than $AUD1000 and 19% missed medical appointments or failed to fill prescriptions because they were short of money. While not explored in this study, other research has shown that clinicians are often unaware that their patients’ are facing difficulty managing out-of-pocket costs [25]. Patients can be reluctant to discuss cost pressures with their health care providers because of a perception that there are few viable solutions to the problem [10,24,25]. Clinicians could play a greater role in identifying patients at risk of hardship and linking them into existing social welfare support programs.

This study also raises an important implication for clinical practice. In contrast to the published literature on the quality of life of CKD patients [26], we found no differences in quality of life scores across the different stages of illness. However, patients with hardship had lower quality of life across the three stages of CKD and it was significantly lower for patients receiving dialysis care. These findings highlight the inter-relationship between chronic illness, economic wellbeing and quality of life. Maximising quality of life is an important treatment outcome to minimise morbidity and mortality rates in the management of CKD patients. However, maximising quality of life is also associated with other important economic outcomes such as maintaining employment and independence [27]. The clinical management of CKD should take account of these broader outcomes which are relevant to the overall well-being of patients and their households.

This study has limitations. We achieved a participation rate of 63% and over-sampled patients receiving dialysis care in a community setting. This sample is also drawn from a predominantly low socioeconomic setting. However, comparison of the demographic and socioeconomic characteristics of our sample with the treated incident renal population in Australia and a national census of patients on dialysis shows that our sample is younger, less sick and has a higher proportion in patients still in paid employment (Additional file 1: Table S2). We have likely under-estimated the true economic impact of CKD for patients who are socioeconomically disadvantaged. In addition, the observed non-response is likely related to both outcomes and to hardship status, giving a potential for bias in association. We did not sample non-English speaking participants. The extent of hardship may be even more pronounced in these groups because of inherent reasons, including their ability to access and navigate existing social welfare supports. Finally, we measured hardship at one time point. It is likely that patients employ different coping strategies over time to improve their circumstances. A prospective design would allow assessment of the change in financial circumstances and understand better the direction of causation. In this study, the aim was instead to describe the lived experience of patients in which economic hardship, poor health and other aspects of social disadvantage co-exist. Given that our study population had been receiving dialysis for a mean of five years and were on average 12 years past their diagnosis, our results likely reflect the situation of the established ‘steady state’ for the households affected, demonstrating a need for early interventions to assist households to better cope with the negative economic sequelea of CKD.

## Conclusion

This study found a considerable proportion of patients face hardship and high out-of-pocket spending associated with the care and management of CKD in Australia. This research raises important practice implications for clinicians who could play a greater role in identifying and supporting patients who are experiencing hardship, particularly in settings that rely heavily on out-of-pocket costs to fund CKD treatment, such as in the US. It also provides a basis for further investigation of the additional supports required to better assist households affected by CKD, which would likely also be of benefit to other chronically ill patient populations.

## Competing interest

The authors declare that they have no competing interests.

## Authors’ contributions

BE, SJ, GW and JC conceived of the study and participated in its design and coordination. BE collected all data, conducted data analysis and wrote the first draft of the paper. QL provided statistical expertise and guidance. All authors read and approved the final manuscript.

## Pre-publication history

The pre-publication history for this paper can be accessed here:

http://www.biomedcentral.com/1471-2369/14/5/prepub

## Supplementary Material

Additional file 1: Table S1 Summary of domains included in the study questionnaire. **Table S2.** Comparison of demographic and socio-economic characteristics between the study participants receiving renal replacement therapy with the treated incident renal population in Australia. Click here for file

## References

[B1] Australian Institute of Health and Welfare Health care expenditure on chronic kidney disease in Australia 2009 Canberra: AIHW 117 Cat. no. PHE

[B2] Baboolal K McEwan P Sondhi S The cost of renal dialysis in a UK setting—a multicentre study Nephrol Dial Transplant 2008 23 1982 1989 10.1093/ndt/gfm870 18174268

[B3] Zelmer JL The economic burden of end-stage renal disease in Canada Kidney Int 2007 72 1122 1129 10.1038/sj.ki.5002459 17700643

[B4] Australian Institute of Health and Welfare Health expenditure Australia: 2010–11. Health and welfare expenditure series no. 47. Cat. no. HWE 56 2012 Canberra: AIHW

[B5] Australian Institute of Health and Welfare Dialysis and kidney transplantation in Australia: 1991–2010 2012 Canberra: AIHW Cat. no. PHE 162

[B6] Healthcare Management Advisors Pty Ltd and Victorian Department of Human Services Renal Dialysis Costing and Funding Review. Draft Final Report 2006 Collingwood: Healthcare Management Advisors Pty Ltd Available from: http://docs.health.vic.gov.au/docs/doc/B287F4FEF5FE63ECCA2578E20006ACFA/$FILE/renal-cost-finrep-draft12dec.pdf [cited: 11 December 2011

[B7] Muehrer RJ Schatell D Witten B Factors affecting employment at initiation of dialysis Clin J Am Soc Nephrol 2011 6 489 496 10.2215/CJN.02550310 21393489 PMC3082405

[B8] Alexander GC Casalino LP Meltzer DO Patient-physician communication about out-of-pocket costs JAMA 2003 290 953 958 10.1001/jama.290.7.953 12928475

[B9] Essue B Kelly P Roberts M We can't afford my chronic illness! The out-of-pocket burden associated with managing chronic obstructive pulmonary disease in western Sydney, Australia J Health Serv Res Pol 2011 16 226 231 10.1258/jhsrp.2011.010159 21954233

[B10] Piette JD Heisler M Wagner TH Problems paying out-of-pocket medication costs among older adults with diabetes Diabetes Care 2004 27 384 391 10.2337/diacare.27.2.384 14747218

[B11] Jeon Y-H Essue B Jan S Economic hardship associated with managing chronic illness: a qualitative inquiry BMC Heal Serv Res 2009 9 182 10.1186/1472-6963-9-182 PMC2766369 19818128

[B12] Jan S Essue BM Leeder SR Falling through the cracks: the hidden economic burden of chronic illness and disability on Australian households MJA 2011 196 1 3 10.5694/mja11.11105 22256924

[B13] Hackett ML Glozier NG Jan S Psychosocial Outcomes in StrokE: the POISE observational stroke study protocol BMC Neurol 2009 9 10.1186/1471-2377-9-24 PMC2708124 19519918

[B14] Hackett ML Glozier NS Martiniuk AL Sydney epilepsy incidence study to measure illness consequences: the SESIMIC observational epilepsy study protocol BMC Neurol 2011 11 10.1186/1471-2377-11-3 PMC3025848 21214957

[B15] The EQ-5D: a standardised instrument for use as a measure of health outcome Available from: http://www.euroqol.org/

[B16] Banks E Redman S 45 and Up Study Collaborators Cohort profile: the 45 and up study Int J Epidemiol 2008 5 941 947 17881411 10.1093/ije/dym184 PMC2557061

[B17] Australian Bureau of Statistics 2010 General Social Survey: Summary results 2011 Canberra: ABS. Cat no. 4159.0

[B18] Grootaert C Narayan D Jones VN Measuring Social Capital: an integrated questionnaire. Working paper No. 18 2004 Washington: The World Bank

[B19] de Vos K Zaidi MA Equivalence scale sensitivity of poverty statistics for the member states of the European Community Review of Income and Wealth 1997 43 319 333 10.1111/j.1475-4991.1997.tb00222.x

[B20] Berki SE A look at catastrophic medical expenses and the poor Health Aff (Millwood) 1986 5 138 145 10.1377/hlthaff.5.4.138 3102333

[B21] Gordon EJ Prohaska TR Sehgal AR The financial impact of immunosuppressant expenses on new kidney transplant recipients Clin Transplant 2008 22 738 748 10.1111/j.1399-0012.2008.00869.x 18673373 PMC2592494

[B22] Schoen C Osborn R Squires D How health insurance design affects access to care and costs, by income in eleven countries Health Aff 2010 29 2323 2334 10.1377/hlthaff.2010.0862 21088012

[B23] Hirth RA Greer SL Albert JM Out-Of-Pocket Spending And Medication Adherence Among Dialysis Patients In Twelve Countries Heal Aff 2008 27 89 102 10.1377/hlthaff.27.1.89 18180483

[B24] Hynd A Roughead EE Preen DB The impact of co-payment increases on dispensings of government-subsidised medicines in Australia Pharmacoepidemiology and Drug Safety 2008 17 1091 1099 10.1002/pds.1670 18942671

[B25] Piette JD Heisler M Wagner TH Cost-related medication underuse: do patients with chronic illnesses tell their doctors? Arch Intern Med 2004 164 1749 1755 10.1001/archinte.164.16.1749 15364667

[B26] Liem YS Bosch JL Myriam Hunink MG Preference-based quality of life of patients on renal replacement therapy: a systematic review and meta-analysis Value Health 2008 11 733 741 10.1111/j.1524-4733.2007.00308.x 18194399

[B27] Blake C Codd MB Cassidy A Physical function, employment and quality of life in end-stage renal disease J Nephrol 2000 13 142 149 10858978

