# Supplementary material for: How are patients managing with the costs of care for chronic kidney disease in Australia? A cross-sectional study
Source: BMC Nephrol. 2013 Jan 10;14:5. doi: 10.1186/1471-2369-14-5 (PMC3698195; doi:10.1186/1471-2369-14-5)
Supplement: Additional file 1: Table S1 — Summary of domains included in the study questionnaire. Table S2. Comparison of demographic and socio-economic characteristics between the study participants receiving renal replacement therapy with the treated incident renal population in Australia. [file 1471-2369-14-5-S1.docx]

**Supplementary Results**

Table S1. Summary of domains included in the study questionnaire

| Domains | Variables |
| --- | --- |
| Personal and family information[1] | - Date of birth |
|  | - Sex |
|  | - Indigenous heritage |
|  | - Ethnicity |
|  | - Language spoken at home |
|  | - Marital status |
|  | - Household composition |
| Medical history [2] | - Year of diagnosis |
|  | - Stage of disease (pre-dialysis, dialysis, transplant) |
|  | - Number of transplants |
|  | - Date of current transplant |
|  | - Type of transplant |
|  | - Dialysis history |
|  | - Type of dialysis treatment |
|  | - Mode of transportation to dialysis treatment |
|  | - Co-morbidities |
|  | - Current weight and height |
|  | - Illness perceptions [3] |
|  | - Self-assessed health |
|  | - Quality of life [4] |
| Education and employment [1] | - Highest qualification achieved |
|  | - Current work status |
|  | - Medical related retirement |
|  | - Lifetime occupation |
| Social connection [1,5] | - Number of close friends |
|  | - Access to financial support |
|  | - Number of social contracts in previous week |
|  | - Perception of access to assistance |
|  | - Perception of neighbourhood risk of harm |
|  | - Perception of power to make decisions |
| Need for assistance with activities of daily living | - Personal care, getting around, preparing meals, housework, shopping, household and garden maintenance, medical care |
|  | - Family carer |
|  | - Impact on carer: social, education and employment |
| Household financial situation [6,7] | - Access to financial resources |
|  | - Financial stressors (utility bills; rent and mortgage; car registration; credit cards; medications; medical consultations and tests; health insurance premiums; dental appointments; child care; transport; food) |
|  | - Dissaving actions (savings; moved house; credit; borrowed; assistance from charities; formal and informal loans; sold assets) |
|  | - Out-of-pocket costs (medications; health care professionals; medical tests; transport; supportive assistance; medical equipment and supplies; home modifications; illness-specific diet) |
|  | - Recipient of government assistance |
|  | - Income |
|  | - Private health insurance |
|  | - Perception of financial needs |
|  |  |

Table S2. Comparison of demographic and socio-economic characteristics between the study participants receiving renal replacement therapy with the treated incident renal population in Australia

|  | **A**  **Study participants on RRT (n=224)** | **B**  **Population estimates for all patients receiving RRT in Australia** | ***P-value*** |
| --- | --- | --- | --- |
| *Demographic and illness characteristics* |  | n=13,918[24, 26] |  |
| Age |  |  |  |
| >25 years (ref) | 5 (2) | 465 (3) | 0.48 |
| 25-44 years | 31 (14) | 2013 (14) | 0.62 |
| 45-64 years | 105 (47) | 5115 (37) | 0.002 |
| 65-84 years | 80 (36) | 6027 (43) | 0.04 |
| >85 years | 1 (0.5) | 298 (2) | 0.14 |
| Gender (Females) | 95 (43) | 5489 (39) |  |
| Co-morbid conditions |  |  |  |
| Diabetes | 92 (41) | 6015 (43) | 0.57 |
| Lung disease | 7 (3) | 2247 (16) | 0.0001 |
| CVD | 78 (35) | 7747 (56) | 0.0001 |
| Quality of life |  |  |  |
| Haemodialysis | 0.63 (0.59-0.67) | 0.56 (0.49-0.62) | 0.0003 |
| Peritoneal dialysis | 0.71 (0.66-0.76) | 0.58 (0.50-0.67) | 0.0001 |
| Transplant | 0.66 (0.55-0.77) | 0.81 (0.72-0.90) | 0.008 |
| *Socio-economic characteristics* |  | n=3,250[27] |  |
| Home ownership (without a mortgage) | 151 (67) | 2107 (65) | 0.48 |
| Employment status |  |  |  |
| Employed (full-time or part-time) | 43 (19) | 299 (9) | 0.0001 |
| Retired or not looking for work | 179 (81) | 2428 (75) | 0.10 |
| Private health insurance | 78 (35) | 828 (25) | 0.003 |
|  |  |  |  |

Data are presented as either mean (95% CI) or count (%=proportion).

Dialysis patients and transplant recipients only (n=224, missing demographic data for 2 participants)

*P*-value assesses significant differences between the study population (column A) and the treated incident renal population in Australia (column B).

**References**

1. Banks E, Redman S, Jorm L, et al. **Cohort profile: the 45 and up study**. *Int J Epidemiol*. 2008;**37**:941-7.
2. Kidney Health Australia. **Consumer Perspectives on Dialysis: First National Census**. Melbourne: Kidney Health Australia, 2011 [date accessed: January 2011]. Available from: http://www.kidney.org.au//LinkClick.aspx?fileticket=JfvirIbNWxA%3d&tabid=618&mid=854

1. Broadbent E, Petrie KJ, Main J, Weinman J: **The brief illness perception questionnaire**. *J Psychosomat Res* 2006;**60**:631-637.
2. **The EQ-5D: a standardised instrument for use as a measure of health outcome**. Rotterdam: The EuroQoL Group; 2010.
3. Grootaert C, Narayan D, Jones VN, Woolcock M: **Measuring Social Capital: an integrated questionnaire. Working paper No. 18**. Washington: The World Bank; 2004.
4. Australian Bureau of Statistics. **2010 General Social Survey: Summary results**. 4159.0. Canberra: ABS, 2011. [date accessed June 2012]. Available from: http://www.ausstats.abs.gov.au/Ausstats/subscriber.nsf/0/D0B6CB77DE0BF677CA25791A00824C41/$File/41590_2010.pdf
5. Prah Ruger J. **An Alternative framework for analysing financial protection in health**. *PLoS Med* 2012,**9**: e1001294. doi:10.1371/journal.pmed.1001294
